# Independent prognostic importance of endothelial activation and stress index (EASIX) in critically ill patients with heart failure: modulating role of inflammation

**DOI:** 10.3389/fmed.2025.1560947

**Published:** 2025-05-01

**Authors:** Fang Yin, Kai Wang

**Affiliations:** ^1^Department of Infectious Diseases, The Affiliated Yongchuan Hospital of Chongqing Medical University, Chongqing, China; ^2^Department of Cardiology, The Second Affiliated Hospital of Chongqing Medical University, Chongqing, China

**Keywords:** EASIX, endothelial activation and stress index, heart failure, intensive care unit, mortality

## Abstract

**Background:**

The connection between endothelial activation and stress index (EASIX) and risk of mortality in critically ill patients with heart failure (HF) remains unclear. This research sought to explore this relationship.

**Methods:**

MIMIC-IV database (version 3.1) was utilized to provide clinical data. Due to the non-normal distribution, EASIX was logarithmic. An optimal cut-off value for log2(EASIX) was determined to serve as an indicator of mortality risk under the maximally selected rank statistics. Kaplan-Meier survival analysis and Cox regression models were used to assess the link between log2(EASIX) and mortality within 1 year. Subgroup analyses were performed to ascertain the prognostic impact of log2(EASIX) in various patient groups. Mediation analysis was employed to uncover and elucidate causal pathways connecting log2(EASIX) to mortality.

**Results:**

It encompassed 7,901 patients. According to the Kaplan-Meier curves, increased log2(EASIX) levels correlated with a higher likelihood of all-cause mortality (*p* < 0.001). Cox models and subgroup analyses further revealed that groups with high log2(EASIX) levels exhibited a greater mortality risk than those with lower levels (hazard ratio (HR): 1.62, 95% CI: 1.47–1.78), a trend that persisted across most subgroups, with the exception of varying levels of APS III, body mass index, white blood cell counts, or albumin (p for interaction < 0.05 for all). Subsequent mediation analysis suggested that blood urea nitrogen and red cell distribution width partially mediated the relationship between log2(EASIX) and mortality with 17.3% and 36.5% of the mediating effect.

**Conclusion:**

It found an independent association between elevated log2(EASIX) levels and a higher risk of 1 year all-cause mortality in ICU patients suffering from HF, with a stronger effect observed in patients with low levels of APS III or white blood cell counts, or high levels of body mass index or albumin. This association may be partially mediated by blood urea nitrogen and red cell distribution width.

## Introduction

Heart failure (HF) continues to be a major health concern, characterized by persistently high rates of morbidity and mortality (1). From 1990 to 2019, the global prevalence of HF increased by 106.3%, reaching 56.2 million cases in 2019 (2). The complex interplay of comorbidities, the severity of HF, and multi-organ dysfunction necessitates advanced care for critically ill HF patients in intensive care units, posing substantial challenges for comprehensive management. Risk stratification holds promise in aiding clinicians to make informed decisions, potentially reversing the current adverse trends (3). Despite the introduction of risk prediction tools, their clinical utility is often limited by the instability of their predictive performance and the complexity of certain models (4). Therefore, there is an urgent need to identify additional non-invasive risk factors to address these challenges. Furthermore, it is crucial to identify available markers, considering the regional disparities in healthcare resources.

Recently, the role of cardiac microvascular endothelial injury in HF has garnered increased attention (5). Endothelial cells are integral to the pathophysiology of HF, playing a pivotal role in maintaining vascular homeostasis. Dysfunction of these cells can lead to impaired vasodilation, inflammation, and thrombosis, which may further exacerbate endothelial damage (6). The endothelial activation and stress index (EASIX) is composed of lactate dehydrogenase (LDH), creatinine, and platelet levels, initially formulated to determine the severity of endotheliopathy following stem cell transplantation (7). Previous studies have established that elevated EASIX levels were correlated with an increased risk of mortality in patients post-allogeneic stem cell transplantation, a condition linked to thrombotic microangiopathy resulting from endothelial dysfunction (8, 9). LDH, a component of EASIX, is released from endothelial cells, platelets, and leukocytes when the vascular endothelium is compromised, leading to elevated levels. In patients with elevated EASIX levels (severe endothelial damage), elevated creatinine levels may indicate a relationship between endothelial damage and renal function injury, while decreased platelet levels may be due to endothelial damage and complement activation. Exposure to collagen and increased levels of von Willebrand factor and tissue factors due to endothelial injury promote platelet over-activation and aggregation (10). Subsequent researches have increasingly identified EASIX as a possible indicator for patients with sepsis (11), severe liver disease (12), acute pancreatitis (13), or traumatic brain injury (14). This has led researchers to propose that EASIX may serve as a global indicator of endothelial cell dysfunction, prompting further investigations into its possible association with cardiovascular diseases. Notably, studies have demonstrated a strong correlation between elevated EASIX levels and poor outcomes in patients with coronary artery disease (15), including acute myocardial infarction (16). However, the relationship between EASIX, as a cost-effective marker, and the prognosis of patients with severe HF remains unexplored.

Consequently, the link between endothelial damage and prognosis in critically ill HF patients requires further examination. To address this gap, we conducted a comprehensive analysis using the MIMIC-IV database, encompassing a substantial cohort of patients. Moreover, subgroup analyses were carried out to identify the optimal population for EASIX application.

## Materials and methods

### Study participants

This group was sourced from MIMIC-IV version 3.1, including data from 2008 to 2022, and has been approved for Kai Wang (ID: 64734176). The Human Institutional Review Board at Beth Israel Deaconess Medical Center approved the study, which involved a secondary analysis of anonymized data that cannot be used to identify individuals. As a result, the need to get informed consent from patients and conduct an additional ethical review was waived.

**FIGURE 1:**
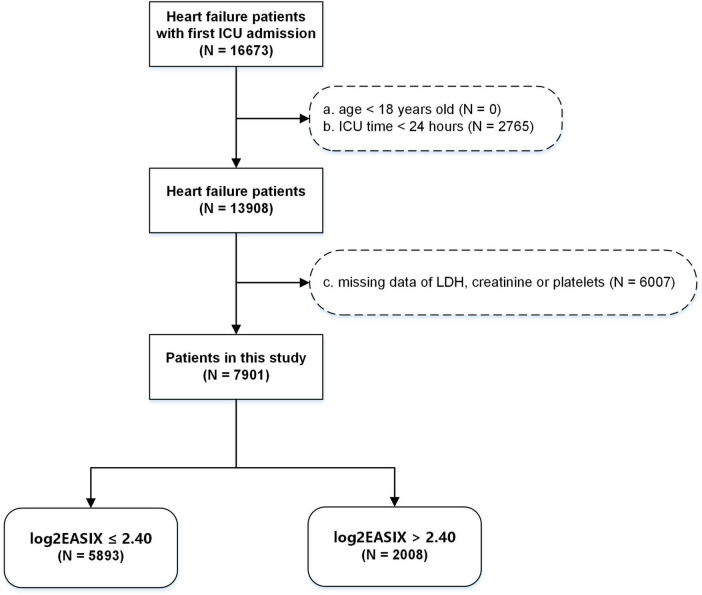
Flow chart of study population inclusion.

Diagnoses were based on the International Classification of Diseases. All patients with HF with ICU admission for the first time were enrolled. The individuals under 18 years old, with ICU stay of less than 24 h, or without data of serum LDH, serum creatinine or platelets were excluded (Figure 1).

### Data collection

Within 24 h of ICU admission, the initial measurement was used to extract clinical data. Any data with more than 20% missing values were removed, with exception of the height data (40% of missing) calculating body mass index (BMI). The data included age, gender, body weight and height, heart rate, saturation of peripheral oxygen (SpO2), blood pressure, respiratory rate, comorbidities (chronic kidney disease (CKD), acute kidney injury (AKI), sepsis, diabetes, hypertension, COVID-19), laboratory data (platelets, red cell distribution width (RDW), hemoglobin, white blood cell (WBC), total bilirubin (TBil), albumin, alanine aminotransferase (ALT), aspartate aminotransferase (AST), creatinine, blood urea nitrogen (BUN), LDH), scores of illness severity (SOFA and APS III), inhibitors of renin angiotensin aldosterone system (RAAS inhibitors covering angiotensin receptor-neprilysin inhibitors, angiotensin receptor blockers, or angiotensin converting enzyme inhibitors), β blockers, steroids, continuous renal replacement therapy (CRRT), and mechanical ventilation. Sepsis was defined as compliance with the diagnostic criteria for sepsis 3.0 (17).

The exposure was EASIX obtained by this formula, LDH level (U/L) × creatinine level (mg/dL) / platelets (10∧9/L) (16). The endpoint was defined as 1 year mortality from all causes.

### Statistical analysis

To address the issue of missing data, the multiple imputation method was employed under the assumption of missing-at-random (MAR) data. Specifically, variables with less than 20% missingness were subjected to multiple imputation via chained equations (MICE) using the *mice* package, resulting in the generation of five complete datasets through 50 iterations, followed by the application of Rubin’s rules for pooled estimates.

Continuous variables were expressed as mean and standard deviation or median and quartiles, while categorical variables were presented as number and percentage. EASIX followed a skewed distribution, so it was transformed using a base-2 logarithm [log2(EASIX)] so that it satisfied the application conditions of generalized linear regression (16). All were conducted in R (version 4.4.1), with a significance threshold set at *p* < 0.05.

Firstly, the optimal cut-off value of log2(EASIX) was obtained for early warning of mortality risk, under the maximally selected rank statistics using the *survminer* package. Kaplan-Meier curves stratified by the low- or high- level of log2(EASIX) and Cox models adjusted for confounders to minimize confounding bias evaluated the mortality risk [hazard ratio (HR)]. The selection of covariates was informed by prior research and clinical practice expertise, rather than data-driven methods (18), including gender and age as demographics; hypertension, diabetes, sepsis, AKI, CKD and COVID-19 as comorbidities; hemoglobin and WBC as markers of anemia and inflammation; AST, TBil, albumin, creatinine and LDH as liver and kidney function; β blockers, RAAS inhibitors, CRRT and ventilation as therapy-related factors; as well as SOFA and APS III as disease severity. Notably, the mediating variables were excluded out of the confounders. Variance inflation factors were calculated to identify multicollinearity, with values over five suggesting possible multicollinearity among the variables. To confirm the reliability of the analysis results, the relative ratios (RR) for log2(EASIX) were calculated using modified Poisson regression, with 1 year all-cause mortality as outcome.

Then, subgroup analysis and interaction testing were conducted by adjusted Cox models to evaluate the HR across various patients. Specifically, recognizing that APS III, BMI, WBC and albumin were inherently continuous, we incorporated them into analysis visualized using restricted cubic splines (RCS) curves (19), to evaluate the continuous interaction between APS III, WBC or albumin and log2(EASIX) in all patients, and between BMI and log2(EASIX) in patients with available body weight and height data. These were executed using the *interactionRCS* package.

Subsequently, mediation analysis was employed to uncover causal pathways connecting the log2(EASIX) to mortality (20), providing insights into strategies for improving patient outcomes. The proportion of indirect effects was calculated as the ratio of the indirect effects to the total effects. Additionally, the mediation effect analyses were conducted between log2(EASIX) and variables including platelets, creatinine, and LDH. When both the mediating and direct effects were statistically significant, and the mediating effect was opposite in sign to the direct effect, then it indicated a masking effect. This masking effect was quantified as the ratio of the absolute value of the mediating effect to that of the direct effect. These was executed using the *lavaan* package.

Finally, to further improve the robustness of analysis, all baseline information was controlled using inverse probability of treatment weighting (IPTW) and probability score matching (PSM). PSM- or IPTW- adjusted Kaplan-Meier survival curves and Pepe-Flemming test were used to compare the mortality risk between patients with low- and high-level log2(EASIX) (21). This was conducted using the adjustedCurves package.

## Results

### Patient characteristics

**FIGURE 2:**
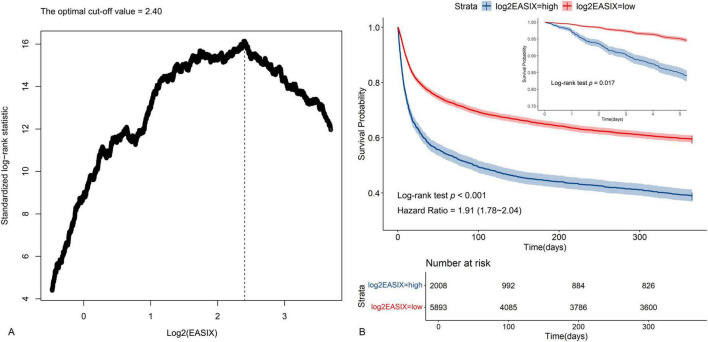
Identification of optimal cutoff value of log2(EASIX) and plotting for the corresponding Kaplan-Meier curves. **(A)** The optimal cutoff value of log2(EASIX) as an warning indicator of mortality risk was determined using the maximally selected rank statistics. **(B)** Kaplan-Meier curves estimated the probability of survival by the levels of log2(EASIX).

All variables entered into the analysis had missing values of less than 20% (Supplementary Figure 1). Totally, the study included 7,901 patients from MIMIC-IV (Table 1). The optimal cut-off value of 2.40 for log2(EASIX) to assess the risk of all-cause mortality in this population (Figure 2A). Among these patients, those with high levels of log2(EASIX) exhibited increased levels of RDW, liver damage and kidney damage, SOFA and APS III, higher proportion of hypertension, diabetes, AKI, CKD and CRRT, decreased levels of blood pressure, hemoglobin, platelets, albumin. Mortality occurred in 45.8% (3,622/7,901) of patients. The log2(EASIX) levels were elevated in patients who were obese or experienced mortality (Supplementary Figure 2). Patients with higher log2(EASIX) values exhibited increased mortality rates of 40.6% and 61.3% (*p* < 0.001).

**TABLE 1:** Baseline information according to the levels of log2(EASIX).

Characteristic	Overall *n* = 7,901	Low (≤ 2.40) *n* = 5,893	High (> 2.40) *n* = 2,008	*P*-value
Age (years)	72.16 ± 13.51	72.61 ± 13.50	70.84 ± 13.45	< 0.001
Female	4,503 (57.0%)	3,183 (54.0%)	1,320 (65.7%)	< 0.001
Respiratory rate (/min)	20.42 ± 6.39	20.37 ± 6.33	20.58 ± 6.56	0.210
Heart rate (/min)	89.90 ± 21.44	90.10 ± 21.44	89.34 ± 21.43	0.172
Systolic blood pressure (mmHg)	119.15 ± 25.45	120.27 ± 25.00	115.86 ± 26.46	< 0.001
Diastolic blood pressure (mmHg)	66.00 (55.00, 78.00)	67.00 (55.00, 79.00)	64.00 (53.00, 77.00)	< 0.001
Saturation of peripheral oxygen (%)	97.00 (94.00, 100.00)	97.00 (94.00, 100.00)	97.00 (94.00, 100.00)	0.787
Hypertension	3,146 (39.8%)	2,095 (35.6%)	1,051 (52.3%)	< 0.001
Diabetes	1,752 (22.2%)	1,157 (19.6%)	595 (29.6%)	< 0.001
Chronic kidney disease	1,963 (24.8%)	1,199 (20.3%)	764 (38.0%)	< 0.001
Acute kidney injury	6,306 (79.8%)	4,517 (76.7%)	1,789 (89.1%)	< 0.001
COVID-19	171 (2.2%)	114 (1.9%)	57 (2.8%)	0.016
White blood cell (10∧9/L)	11.10 (7.90, 15.70)	11.10 (8.00, 15.60)	11.10 (7.40, 16.10)	0.191
Hemoglobin (g/dL)	10.29 ± 2.31	10.43 ± 2.28	9.89 ± 2.34	< 0.001
Red cell distribution width (%)	15.90 ± 2.55	15.68 ± 2.44	16.52 ± 2.75	< 0.001
Platelets (10∧9/L)	190.00 (136.00, 259.00)	207.00 (155.00, 277.00)	134.00 (85.00, 193.00)	< 0.001
Alanine aminotransferase (U/L)	25.00 (15.00, 57.00)	23.00 (14.00, 45.00)	41.00 (19.00 164.25)	< 0.001
Aspartate aminotransferase (U/L)	39.00 (23.00 86.00)	34.00 (22.00 63.00)	75.00 (34.00, 303.50)	< 0.001
Albumin (g/dL)	3.07 ± 0.59	3.10 ± 0.58	2.99 ± 0.61	< 0.001
Total bilirubin (mg/dl)	0.70 (0.40, 1.20)	0.60 (0.40, 1.00)	0.80 (0.50, 1.70)	< 0.001
Creatinine (mg/dl)	1.30 (0.90, 2.20)	1.10 (0.80, 1.60)	2.70 (1.70, 4.60)	< 0.001
Blood urea nitrogen (mg/dl)	29.00 (19.00, 47.00)	25.00 (17.00, 39.00)	47.00 (31.00, 74.00)	< 0.001
lactate dehydrogenase (U/L)	297.00 (223.00, 431.00)	270.00 (210.00, 361.00)	472.00 (307.75, 898.50)	< 0.001
SOFA	5.94 ± 3.54	5.08 ± 3.09	8.48 ± 3.56	< 0.001
APS III	52.24 ± 20.25	48.35 ± 18.12	63.66 ± 21.80	< 0.001
βblockers	5,717 (72.4%)	4,384 (74.4%)	1,333 (66.4%)	< 0.001
Inhibitors of renin angiotensin aldosterone system	2,429 (30.7%)	2,003 (34.0%)	426 (21.2%)	< 0.001
Steroids	2,144 (27.1%)	1,512 (25.7%)	632 (31.5%)	< 0.001
Continuous renal replacement therapy	774 (9.8%)	268 (4.5%)	506 (25.2%)	< 0.001
Ventilation	6,889 (87.2%)	5,134 (87.1%)	1,755 (87.4%)	0.746
1 year mortality	3,622 (45.8%)	2,392 (40.6%)	1,230 (61.3%)	< 0.001

### Kaplan-Meier survival curve

The analysis of Kaplan-Meier survival curves was employed to evaluate the overall survival rate for these two groups categorized by log2(EASIX). Individuals with higher log2(EASIX) levels demonstrated a notably worse prognosis (Log-rank test *p* < 0.001), and the non-adjusted initial HR was 1.91 (95% CI: 1.78–2.04) (Figure 2B). In particular, there was a statistically significant difference in outcome events between the two groups from day 5 onwards (Log-rank test *p* = 0.017) (Figure 2B and Supplementary Figure 3).

### Risk ratios for all-cause mortality

Upon adjusting for potential confounders, all the variance inflation factors were below five, which did not support the existence of multicollinearity (Supplementary Figure 4). The presence of elevated log2(EASIX) was independently associated with high all-cause mortality (HR: 1.62, 95% CI: 1.47–1.78). For each unit increasement in log2(EASIX), the risk increased by 28% (HR: 1.28, 95% CI: 1.23∼1.33) (Table 2). The analysis of Model 0, 1, and 2 showed the consistent results.

**TABLE 2:** Cox models for the association between log2(EASIX) and all-cause mortality.

log2(EASIX)	Case/total	Model 0	Model 1	Model 2	Model 3
		**Hazard Ratio**			
Low (≤ 2.40)	5,893/7,901	Reference	Reference	Reference	Reference
High (> 2.40)	2,008/7,901	1.91 (1.78, 2.04)	1.86 (1.73, 2.00)	1.74 (1.59, 1.91)	1.62 (1.47, 1.78)
Each log2(EASIX) unit increase	–	1.18 (1.16, 1.20)	1.18 (1.16, 1.21)	1.32 (1.27, 1.37)	1.28 (1.23, 1.33)

Model 0: log2(EASIX) was included; Model 1: age, gender, diabetes, hypertension, sepsis, AKI, CKD and COVID-19 were adjusted; Model 2: WBC, hemoglobin, platelets, AST, TBil, albumin, creatinine, LDH, β blockers, RAAS inhibitors, ventilation, and CRRT and were additionally adjusted; Model 3: SOFA and APS III scores were additionally adjusted.

In accordance with the Cox analysis, modified Poisson regression demonstrated that high level of log2(EASIX) remained similarly associated with the elevated risk of mortality (RR: 1.28, 95% CI: 1.21–1.36), with the RR of 1.13 (95% CI: 1.11–1.16) for each unit increasement) (Table 3).

**TABLE 3:** Modified Poison regression models for the association between log2(EASIX) and all-cause mortality.

log2(EASIX)	Case/total	Model 0	Model 1	Model 2	Model 3
		**Relative risk for 1 year mortality**			
Low (≤ 2.40)	5,893/7,901	Reference	Reference	Reference	Reference
High (> 2.40)	2,008/7,901	1.51 (1.44, 1.58)	1.46 (1.39, 1.53)	1.33 (1.25, 1.41)	1.28 (1.21, 1.36)
Each log2(EASIX) unit increase	–	1.11 (1.10, 1.13)	1.11 (1.09, 1.12)	1.15 (1.12, 1.18)	1.13 (1.11, 1.16)

Model 0: log2(EASIX) was included; Model 1: age, gender, diabetes, hypertension, sepsis, AKI, CKD and COVID-19 were adjusted; Model 2: WBC, hemoglobin, platelets, AST, TBil, albumin, creatinine, LDH, β blockers, RAAS inhibitors, ventilation, and CRRT and were additionally adjusted; Model 3: SOFA and APS III scores were additionally adjusted.

**FIGURE 3:**
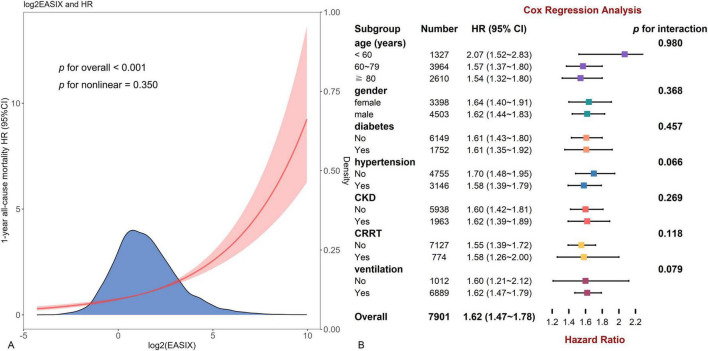
The restricted cubic spline curve for all patients and forest plot for subgroup analysis. **(A)** Restricted cubic spline curve of the log2(EASIX) and hazard ratios in patients with heart failure (HF). **(B)** Subgroup analysis by age, gender, diabetes, hypertension, chronic kidney disease, continuous renal replacement therapy region, and ventilation.

Additionally, as log2(EASIX) levels increased, the risk of all-cause mortality rose linearly, highlighting its robustness (*p* for non-linear = 0.350) (Figure 3A).

### Subgroup analysis

**FIGURE 4:**
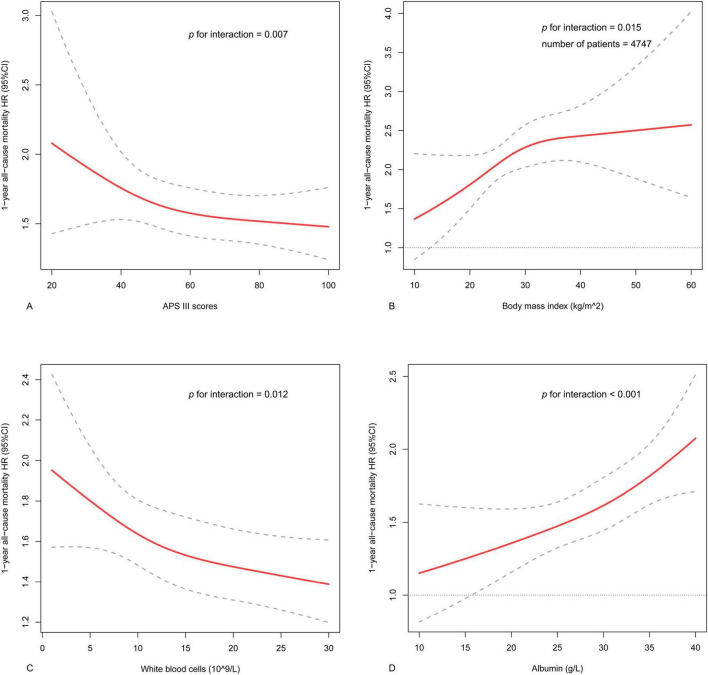
The interactive restricted cubic spline curves for subgroup analysis. **(A)** The analysis in various levels of APS III in the whole population. **(B)** The analysis in various levels of body mass index in part patients with data of body weight and height. **(C)** The analysis in various levels of white blood cell counts in the whole population. **(D)** The analysis in various levels of albumin in the whole population.

Subgroup analysis was conducted along with an interaction analysis. This observed positive relationship was consistent across most subgroups, including age, gender, diabetes, hypertension, CKD, CRRT and ventilation (*p* for interaction > 0.05 for all) (Figure 3B). Dissimilarly, the association was stronger in patients with relatively low levels of APS III (*p* for interaction = 0.007) or WBC (*p* = 0.012), or high BMI (*p* = 0.015) or albumin (*p* < 0.001) (Figures 4A–D).

### Mediating effects analysis

**FIGURE 5:**
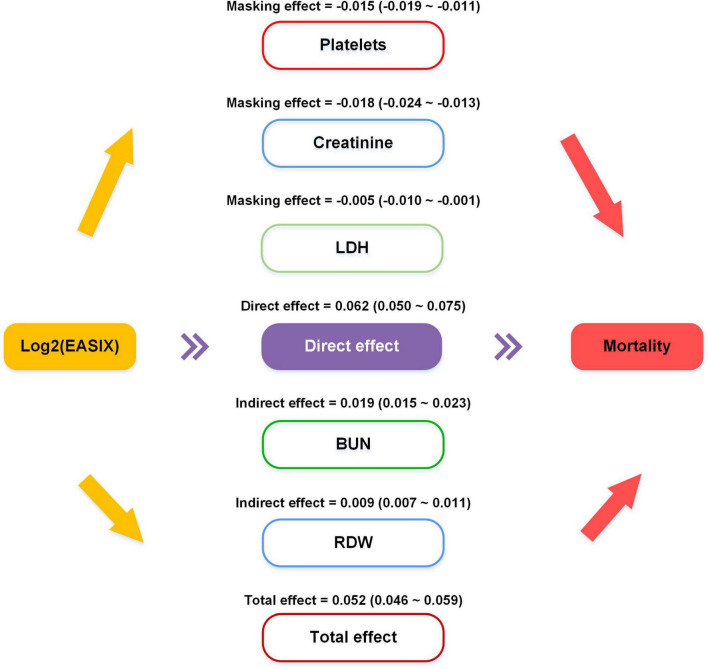
Causal mediation analysis results. The indirect effect stands for average causal mediation effects of log2(EASIX) on the mortality mediated through red cell distribution width and blood urea nitrogen. Platelets, creatinine, and lactate dehydrogenase accounted for 24.2%, 29.0%, and 8.1% of the masking effect. Despite this partial masking, log2(EASIX) significantly contributed to the total effect.

The mediation effect analyses were carried out between log2(EASIX) and RDW and BUN. The results indicated the medication between log2(EASIX) and mortality risk. Specifically, RDW and BUN accounted for 17.3% and 36.5% of the mediating effect in this link (Figure 5). Meanwhile, considering that steroid hormones may cause an increase in BUN levels, which potentially affects the mediating effect of BUN, further mediating effect analyses included steroid hormones as covariates. The results of the analysis showed that BUN retained its role as a mediating variable after adjusting for steroids (Supplementary Figure 5). Additionally, platelets, creatinine and LDH accounted for 24.2%, 29.0%, and 8.1% of the masking effect in this link. Notably, despite partial masking of log2(EASIX)’s direct effect on primary outcomes, it still contributed significantly to the total effect.

### Sensitivity analysis

The PSM and IPTW were employed to achieve balance across all baseline variables presented in Table 1. Subsequently, the Kaplan-Meier curves adjusted using PSM or IPTW demonstrated results consistent with the previous section, indicating that patients with higher log2(EASIX) levels experienced a worse prognosis (Pepe-Flemming test, *p* < 0.001) (Supplementary Figure 6).

## Discussion

Based on the extensive cohort, this study conducted a comprehensive investigation into the independent and positive association between log2(EASIX) and all-cause mortality among ICU patients with HF. The analysis revealed a more pronounced effect in patients exhibiting relatively low APS III or WBC, elevated BMI or albumin levels. Additionally, the study explored potential underlying mechanisms driving this association.

Endothelial activation and stress index, a biomarker increasingly recognized for its prognostic capabilities, particularly in conditions characterized by endothelial dysfunction, is calculated using LDH, creatinine, and platelet counts. This marker reflects the extent of endothelial activation and stress, which are pivotal in the pathogenesis of some diseases. In the context of sepsis, endothelial damage is a critical factor in disease progression, contributing to multiorgan dysfunction and heightened mortality rates (22). Previous research has demonstrated that EASIX may serve as a valuable prognostic tool for assessing the severity of endothelial damage in septic patients (11). Similarly, in individuals with severe liver disease, particularly those experiencing acute-on-chronic liver failure, endothelial dysfunction is a key factor in disease progression. The study has demonstrated that EASIX exhibited moderate predictive power for 28 days and 3 months mortality in patients with advanced liver disease, and it has been proven effective in excluding early infections and forecasting the necessity for hemodialysis in these patients (12). Furthermore, EASIX has been investigated in cardiac conditions such as myocardial infarction, where it has been validated as a potential biomarker for predicting mortality (16). The systemic endothelial health status, as indicated by EASIX, was crucial in assessing the risk of mortality in patients with coronary artery disease, regardless of the timing of coronary interventions (15). Building on these findings, we examined the relationship between log2(EASIX) levels and mortality risk in critically ill patients with HF. Comprehensive data analyses, including survival analysis, Cox regression models, modified Poisson regression, and subgroup analysis, consistently indicated that patients with elevated log2(EASIX) levels faced a heightened risk of mortality. Importantly, this association was independent of the three components of EASIX - LDH, creatinine, and platelets. Meanwhile, as is well established, NT-pro B-type brain natriuretic peptide (NT-proBNP) is an inactive fragment generated through the cleavage of the precursor protein proBNP when ventricular myocytes are stimulated by pressure or traction on the myocardium. NT-proBNP levels serve as indicators of the burden of ventricular pressure and volumetric load. Differently, EASIX reflects endothelial dysfunction throughout the body (including the myocardium). Therefore, theoretically, EASIX may serve as an effective complement to the existing evaluation system. Furthermore, this above correlation was not influenced by impaired liver and kidney function or the patient’s disease severity score including SOFA and APS III, as confirmed by the statistical analysis accounted for all baseline characteristics with PSM and IPTW. The independent prognostic value serves as the foundational basis for predictive power. In the future, incorporating composite indicators such as EASIX into the existing scoring system may enhance the ability for risk warning. In addition, this study identified that the levels of log2(EASIX) in obese HF patients were elevated compared to those in non-obese counterparts, with the prognostic impact of log2(EASIX) being more pronounced in the obese cohort. Current research underscores that endothelial dysfunction is more severe in obese individuals. One study highlighted elevated serum levels of adhesion molecules such as soluble intercellular adhesion molecule-1, soluble vascular cell adhesion molecule-1, and E-selection in obese individuals with atherosclerosis compared to non-obese controls, suggesting that obesity exacerbates endothelial impairment (23). Obesity-related glomerulopathy, characterized by glomerular hypertrophy and proteinuria, is another condition where endothelial dysfunction is evident, further establishing a direct link between obesity and endothelial impairment in renal tissues (24). In summary, these may be interpreted as organ-specific endothelial lesions. This association further supports the utilization of EASIX as a tool for evaluating endothelial impairment. Consequently, we recommend the application of EASIX to assess the risk of endothelial injury and the risk of all-cause mortality in critically ill patients with HF, particularly among those who are obese.

This study found that the prognostic effect of log2(EASIX) was more significant in patients with low APS scores, low WBC, or high albumin. The APS III primarily emphasizes the assessment of damage to vital organs, including the liver, kidneys, and the respiratory and circulatory systems, with a notable deficiency in the evaluation of endothelial impairment (4). Building upon this focus, the EASIX index underscores the importance of endothelial dysfunction in the management of HF. The application of EASIX facilitates precise risk assessment for patients and contributes to a more comprehensive understanding of the underlying pathology. It is widely recognized that patients with elevated APS III are at a heightened risk of adverse events. However, the EASIX indicator demonstrates a stronger impact in patients with lower APS III, suggesting its efficacy in identifying residual risks of adverse events not captured by APS III alone, highlighting the necessity of tracking EASIX levels, even in patients who were clinically stable or showing improvement, since high EASIX levels continued to be linked with poor survival rates in the following year. Moreover, inflammation, oxidative stress, and microvascular endothelial damage interact synergistically, exacerbating cardiac remodeling and dysfunction in the pathogenesis of HF (25). This study identified that inflammatory markers, specifically WBC and albumin levels, modulate the impact of log2(EASIX) on mortality risk in patients with severe HF. These findings exactly underscore the complex interplay among these factors in the context of HF. Moreover, the results support the preference for the EASIX score in patients exhibiting low APS scores, low WBC, or high albumin levels.

This research emphasized the importance of potential mediating variables between log2(EASIX) and mortality risk, highlighting the necessity of understanding the underlying mechanisms. The results of the intermediary effect analysis indicated that the log2(EASIX) had a direct impact on the outcome, independent of platelet, creatinine, or LDH levels, and the link between log2(EASIX) and disease needed further exploration. Notably, the study revealed that RDW and BUN partially mediate this association. RDW is increasingly recognized as a marker of inflammation and oxidative stress (26). Elevated BUN levels suggest impaired kidney function, which is a known risk factor for increased mortality. The modifying effect of kidney function on the relationship between cadmium exposure and both blood pressure and cardiovascular mortality further highlighted the importance of renal markers such as BUN in understanding mortality risk (27). In the context of HF, the impairment of endothelial function leads to an elevated inflammatory state, while compromised renal function fails to adequately remove cytokines from circulation, thereby exacerbating this inflammatory response (28). Severe inflammation could subsequently cause ongoing damage to both the heart and kidneys. It is generally recognized that the formation of cardiorenal syndrome exacerbates the evolution of HF disease. Mechanisms linking these biomarkers to pathophysiological processes may involve dysregulation of iron metabolism and neurohumoral activation, which are characteristic of both conditions (29). In conclusion, further research should elucidate the mechanistic pathways connecting RDW, BUN, and endothelial injury to develop potential interventions aimed at mitigating these risks and enhancing the management of patients with HF. Notably, while statins are well-known for their lipid-lowering properties, they also possess significant pleiotropic effects, such as reducing inflammation and oxidative stress. Statin-based endothelial prophylaxis has been shown to enhance non-relapse mortality and overall survival in patients undergoing allogeneic hematopoietic stem cell transplantation, particularly those with intermediate endothelial risk (30). In HF patients, statin therapy has been linked to a significant reduction in inflammatory markers, suggesting improved endothelial function (31, 32). In the context of renal failure, statins play a crucial role. Patients with CKD are at an elevated risk of cardiovascular disease, partly due to increased oxidative stress and endothelial dysfunction (33). Statins have been demonstrated to reduce oxidative stress and enhance endothelial function in these patients, potentially decreasing the risk of cardiovascular events. Therefore, understanding these mediating roles is crucial for the development of therapeutic strategies, such as the use of statins, aimed at reducing inflammation and oxidative stress, restoring systemic perfusion, and preserving impaired renal function, particularly in patients exhibiting high levels of inflammation, oxidative stress, and elevated EASIX scores. Therefore, we advocate for prospective studies to yield higher quality evidence regarding the value of EASIX across various HF populations. Especially, it is feasible to prioritize research on EASIX-level-guided interventions targeting endothelial dysfunction, such as statin therapy. We believe that EASIX could contribute to the optimization of secondary prevention strategies for patients with HF.

However, this study had several limitations. Firstly, EASIX was currently considered to reflect systemic endothelial impairment, potentially lacking specificity for assessing the degree of cardiac microvascular endothelial dysfunction. The development of simple and accessible serum markers with organ-specificity may represent a promising direction for future research. Secondly, unmeasured confounding factors may persist despite adjustments for major risk factors. These factors primarily included left ventricular ejection fraction and NT-proBNP, due to limitations in the database, which may pose a potential challenge to the findings of this study. Thirdly, determining whether HF was the primary cause of ICU admission and identifying the specific cause of patient mortality within the MIMIC database presented significant challenges. ICU admissions frequently resulted from critical conditions that impact multiple organ systems, including HF, which often led to multi-organ failure. From the patient’s perspective, all-cause mortality may serve as a more meaningful endpoint. Nonetheless, it was essential to acknowledge the distinct pathophysiological mechanisms that differentiate acute from chronic heart failure, as well as heart failure with reduced versus preserved left ventricular ejection fraction. The potential differential role of the EASIX score across various heart failure subtypes remained an area for further investigation. Additionally, it was crucial to recognize that the findings of this study cannot be generalized to infer an association between the EASIX score and cardiovascular mortality or major adverse cardiovascular events.

## Conclusion

The study found that elevated log2(EASIX) levels were independently associated with an increased risk of 1 year all-cause mortality in ICU patients with HF, with stronger effects observed in patients with relatively low levels of APS III or WBC, or high levels of BMI or albumin. This association may be partially mediated by RDW and BUN.

## Data Availability

The raw data supporting the conclusions of this article will be made available by the authors, without undue reservation.

## Supplementary material

Supplementary Figure 1 Visualization of missing values in study population data.

Supplementary Figure 2 Analysis of the difference in levels of log2(EASIX) between different groups. Comparison of levels of log2(EASIX) in groups with or without mortality, and comparison of levels of log2(EASIX) in groups with or without obese.

Supplementary Figure 3 The statistical tests of the difference in outcome event rates between the two groups with low and high log2 (EASIX) levels. From day 5 onwards, the difference was statistically significant (*p* < 0.05).

Supplementary Figure 4 The variance inflation factors between log2(EASIX) and the covariates in fully multivariable analysis. All the values of the variance inflation factors were below five, and the existence of multicollinearity was not considered.

Supplementary Figure 5 Mediator effect analysis performed under the condition of corrected steroid hormones. BUN continued to function as a mediating variable.

Supplementary Figure 6 The covariate-adjusted Kaplan-Meier curves estimated the probability of survival by the levels of log2(EASIX). **(A)** Kaplan-Meier curves were adjusted for all the baseline covariates in Table1 by probability score matching. **(B)** Kaplan-Meier curves were adjusted for all the baseline covariates in Table1 by inverse probability of treatment weighting.
