# Supplementary figures and images for: Independent prognostic importance of endothelial activation and stress index (EASIX) in critically ill patients with heart failure: modulating role of inflammation
Source: Front Med (Lausanne). 2025 May 1;12:1560947. doi: 10.3389/fmed.2025.1560947 (PMC12078299; doi:10.3389/fmed.2025.1560947)

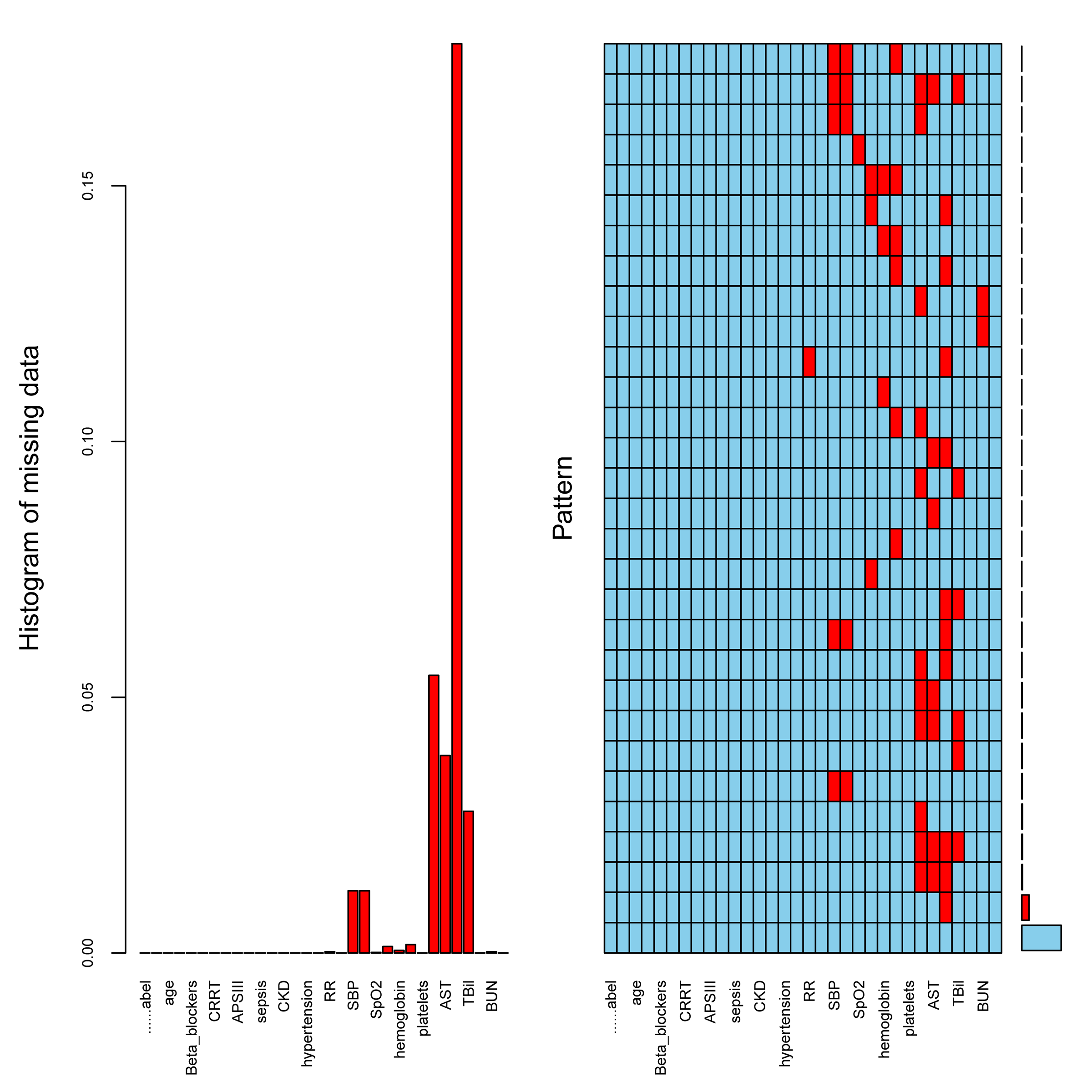

Supplement: Supplementary Figure 1 — Visualization of missing values in study population data. [file Image_1.tiff]

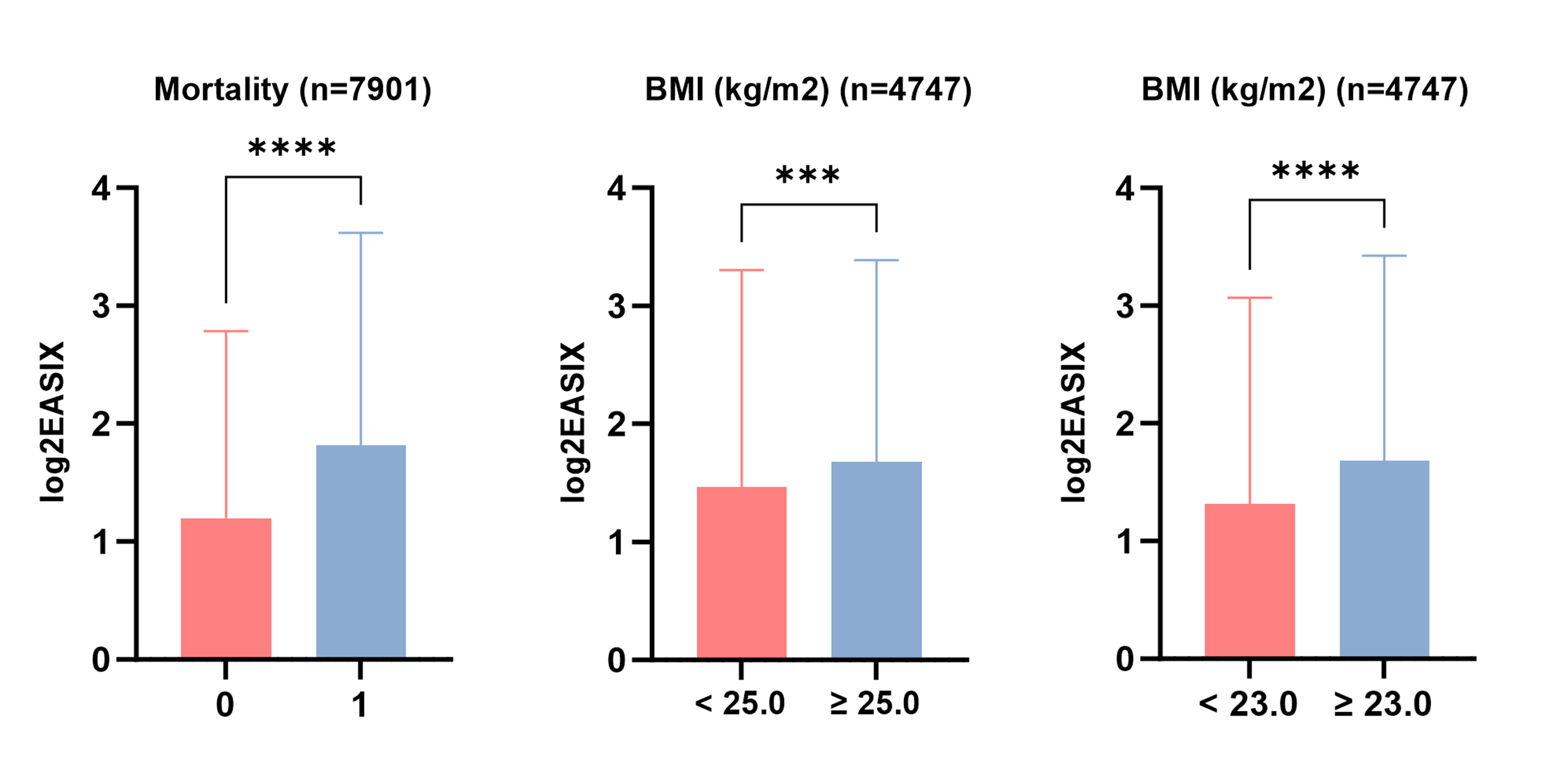

Supplement: Supplementary Figure 2 — Analysis of the difference in levels of log2(EASIX) between different groups. Comparison of levels of log2(EASIX) in groups with or without mortality, and comparison of levels of log2(EASIX) in groups with or without obese. [file Image_2.tiff]

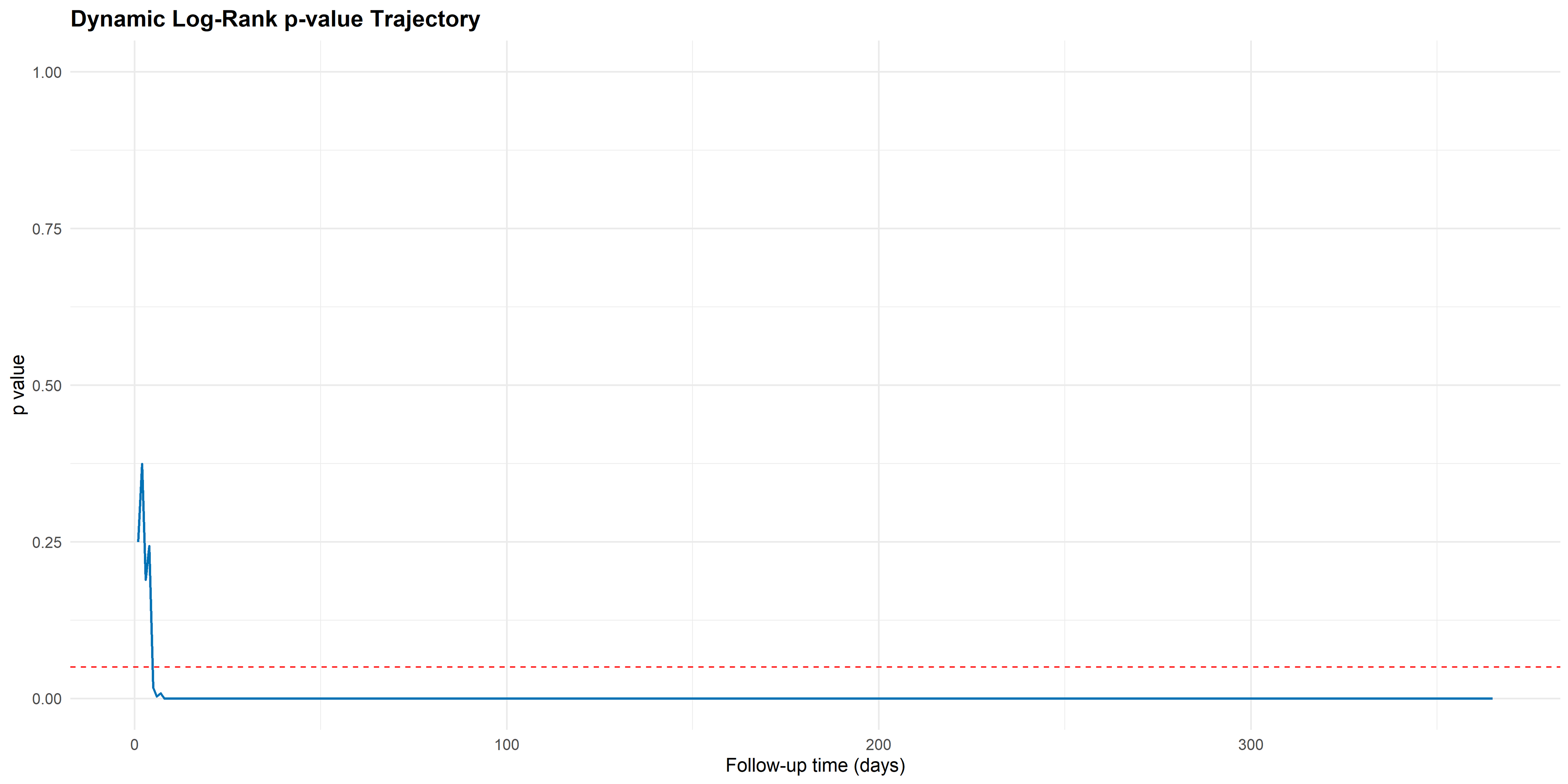

Supplement: Supplementary Figure 3 — The statistical tests of the difference in outcome event rates between the two groups with low and high log2 (EASIX) levels. From day 5 onwards, the difference was statistically significant (p < 0.05). [file Image_3.tiff]

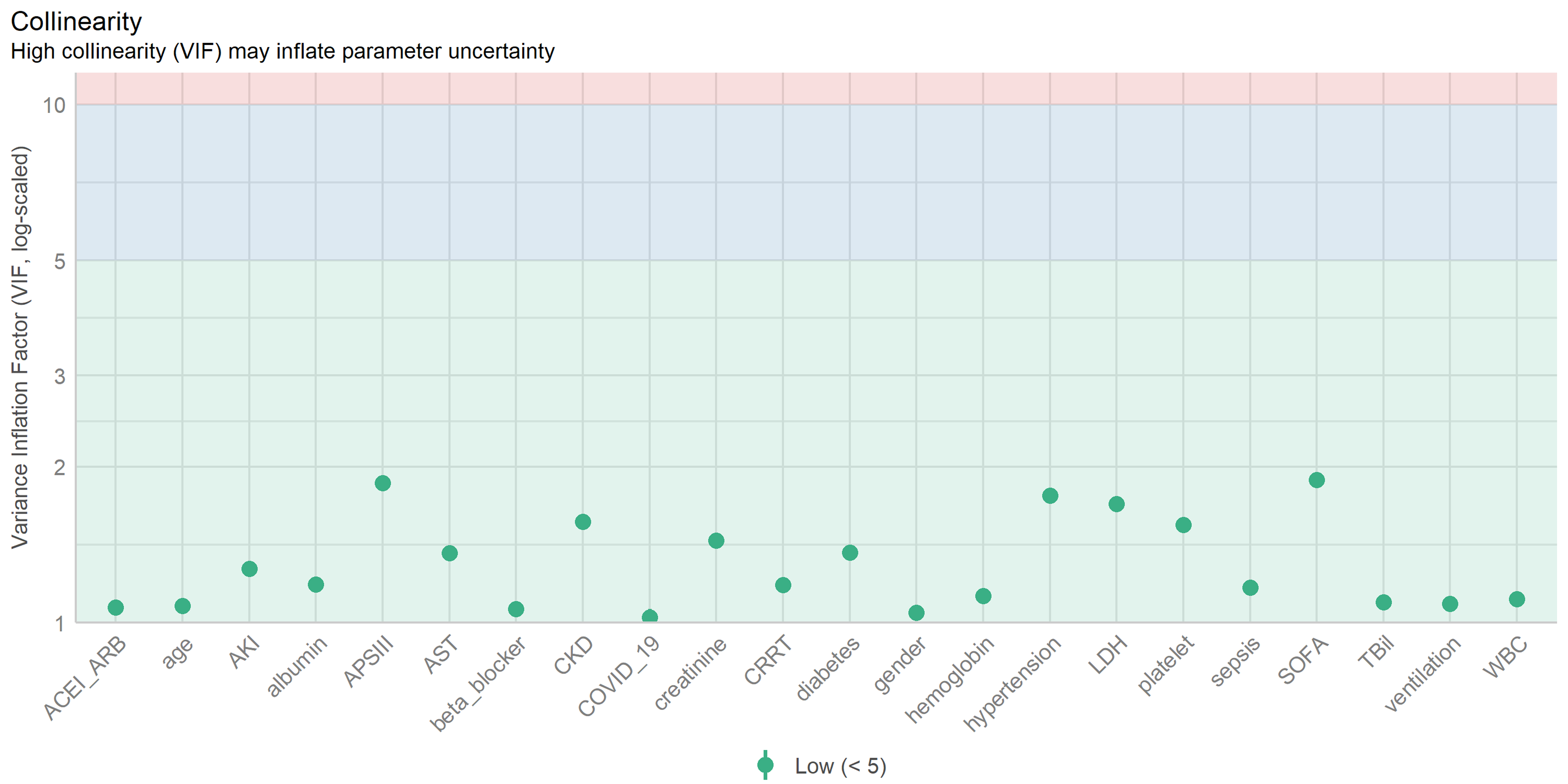

Supplement: Supplementary Figure 4 — The variance inflation factors between log2(EASIX) and the covariates in fully multivariable analysis. All the values of the variance inflation factors were below five, and the existence of multicollinearity was not considered. [file Image_4.tiff]

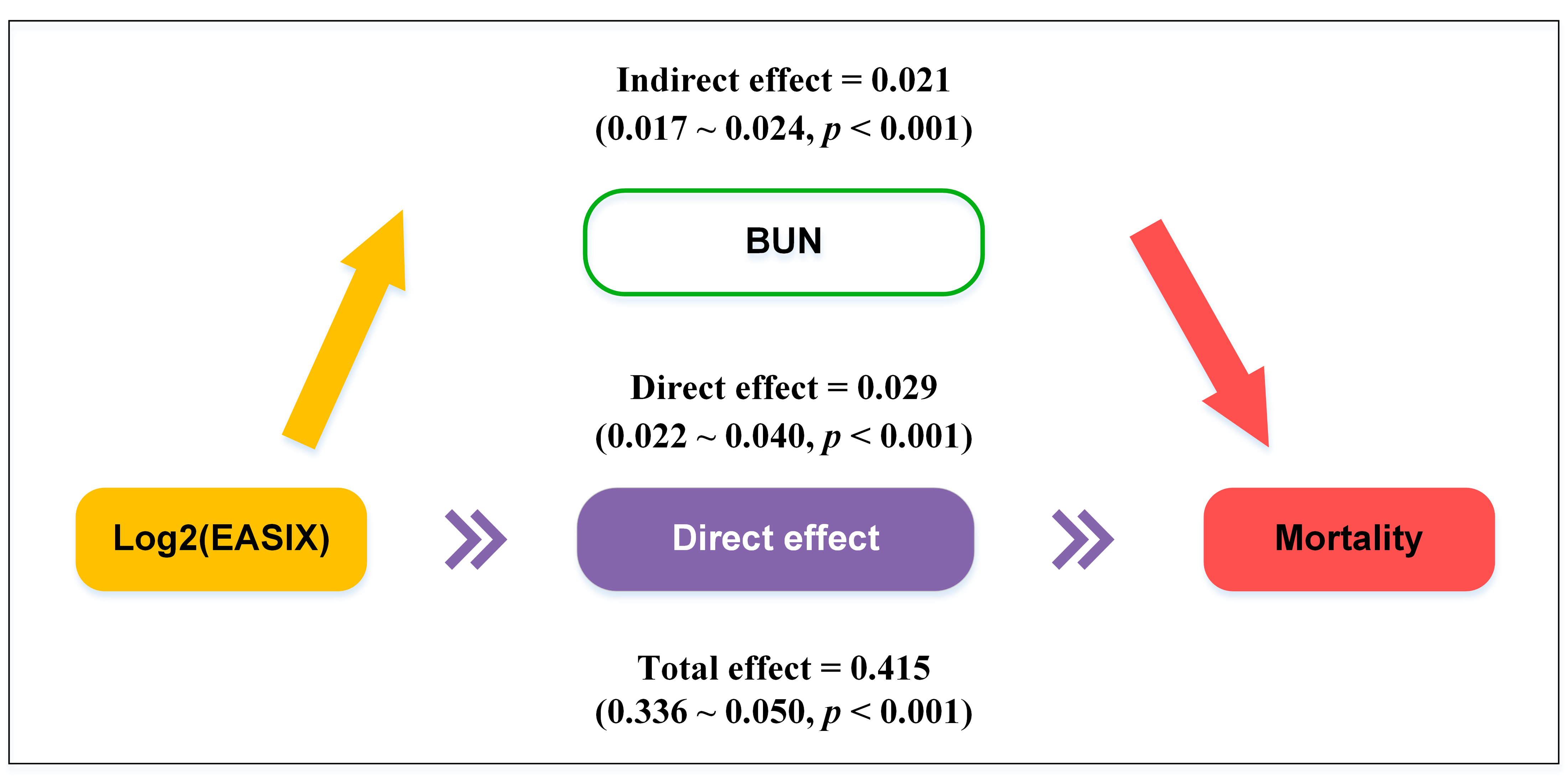

Supplement: Supplementary Figure 5 — Mediator effect analysis performed under the condition of corrected steroid hormones. BUN continued to function as a mediating variable. [file Image_5.tiff]

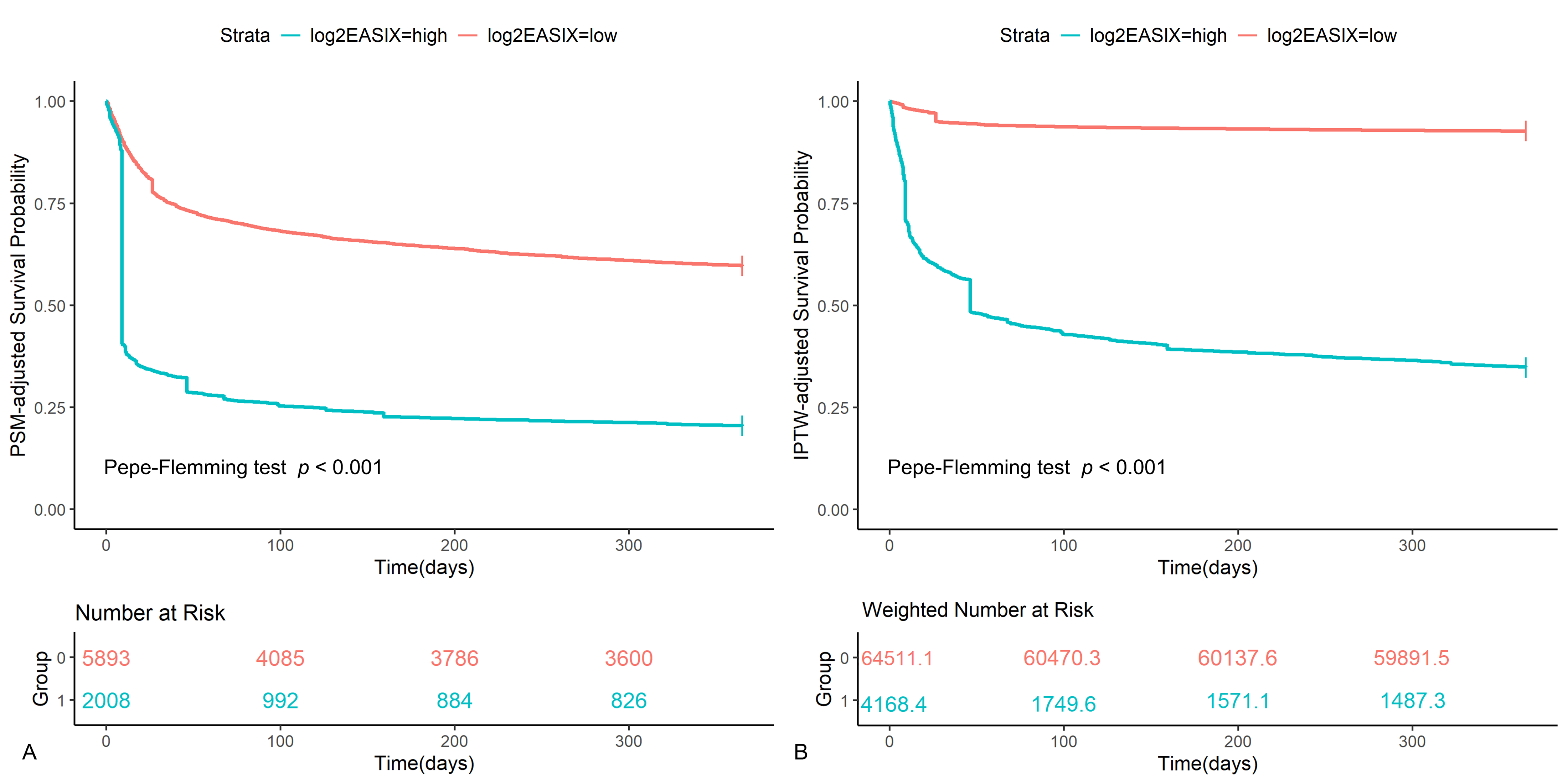

Supplement: Supplementary Figure 6 — The covariate-adjusted Kaplan-Meier curves estimated the probability of survival by the levels of log2(EASIX). (A) Kaplan-Meier curves were adjusted for all the baseline covariates in Table1 by probability score matching. (B) Kaplan-Meier curves were adjusted for all the baseline covariates in Table1 by inverse probability of treatment weighting. [file Image_6.tiff]
